# Facet Engineering in Constructing Lewis Acid-Base Pairs for CO_2_ Cycloaddition to High Value-Added Carbonates

**DOI:** 10.34133/2022/9878054

**Published:** 2022-10-14

**Authors:** Shu Shang, Wei Shao, Xiao Luo, Ming Zuo, Hui Wang, Xiaodong Zhang, Yi Xie

**Affiliations:** ^1^Hefei National Research Center for Physical Sciences at the Microscale, University of Science and Technology of China, Hefei 230026, China; ^2^Institute of Energy, Hefei Comprehensive National Science Center, Hefei 230031, China

## Abstract

Cycloaddition of epoxides with CO_2_ to synthesis cyclic carbonates is an atom-economic pathway for CO_2_ utilization with promising industry application value, while its efficiency was greatly inhibited for the lack of highly active catalytic sites. Herein, by taking BiOX (X = Cl, Br) with layered structure for example, we proposed a facet engineering strategy to construct Lewis acid-base pairs for CO_2_ cycloaddition, where the typical BiOBr with (010) facets expose surface Lewis acid Bi sites and Lewis base Br sites simultaneously. By the combination of *in-situ* diffuse reflectance infrared Fourier transform spectroscopy (DRIFTS) and theoretical calculations, the oxygen atom of the epoxide is interacted with the Lewis acid Bi site to activate the ternary ring, then facilitates the attack of the carbon atom by the Lewis base Br site for the ring-opening of the epoxide, which is the rate-determining step in the cycloaddition reaction. As a result, the BiOBr-(010) with rich surface Lewis acid-base pairs showed a high conversion of 85% with 100% atomic economy in the synthesis of cyclic-carbonates without any cocatalyst. This study provides a model structure for CO_2_ cycloaddition to high value-added long chain chemicals.

## 1. Introduction

The over-use of fossil fuels results in massive emissions of the greenhouse gas (CO_2_), which has a profound impact on the global abnormal climate change and energy crisis [1, 2]. However, from a chemical point of view, carbon dioxide is an important C1 resource due to its inexpensive, abundant, nontoxic, and recyclability [3–5]. The development of approaches to activate and convert CO_2_ into high value-added chemicals, especially in the carbon chain growth reactions of organic synthesis has become a research hotspot nowadays [6–8]. One of the representative applications of carbon dioxide in chemical synthesis is coupling with epoxides to produce cyclic carbonates or polycarbonates, which shows a 100% atomic economy and promising industry application value [2, 9–11]. Cyclic carbonates are notable compounds in industrial production, which are widely used as fuel additives, electrolytes in lithium batteries, aprotic high-boiling polar solvents, degreasers, monomers in the synthesis of polycarbonates, organic synthetic intermediates, and biosynthetic precursors [12]. Of note, conventional industrial production of cyclic carbonates is based on diols with toxic phosgene [13], or using ester exchange, which increases the risk of accidents and unnecessary energy consumption [14]. In contrast, using CO_2_ as C1 feedstock, the direct synthesis of cyclic carbonate from epoxide and carbon dioxide is atomic economy and environmentally-friendly.

As is generally accepted that the ring-opening of the epoxide is the rate-determining step in cycloaddition reaction, it is of great importance to rationally design catalysts that can effectively activate and accelerate the ring-opening of epoxides [15, 16]. In recent years, a large number of homogeneous catalysts have been reported for CO_2_ cycloaddition into cyclic carbonate, including ionic liquids [17], Schiff bases [18], alkali metal halides [19], and quaternary ammonium or phosphonium salts [20], etc. Nevertheless, the development of homogeneous catalysts is greatly limited by the inherent limitations of product separation and catalyst recovery, despite their relatively high catalytic efficiency [21]. Recently, to solving the above shortcomings, heterogeneous catalysts including metal oxides, MOFs were developed for the CO_2_ cycloaddition to various epoxides, while complicated synthetic processes, harsh reaction conditions and requiring the assistance of homogeneous cocatalysts (Tetrabutylazanium bromide (TBAB), etc.) restrict their industrial application [22, 23]. Although the cocatalysts with free halide ions can act as nucleophilic reagents (Lewis base) to attack carbon atoms and accomplish ring opening of epoxide, they would corrode stainless steel at the same time, increasing the risk of chemical leakage [24]. Thus, it is imperative to design efficient catalysts for the CO_2_ cycloaddition with epoxides under relatively mild conditions and non-use of cocatalysts, in particular containing free halide ions.

**Scheme 1:**
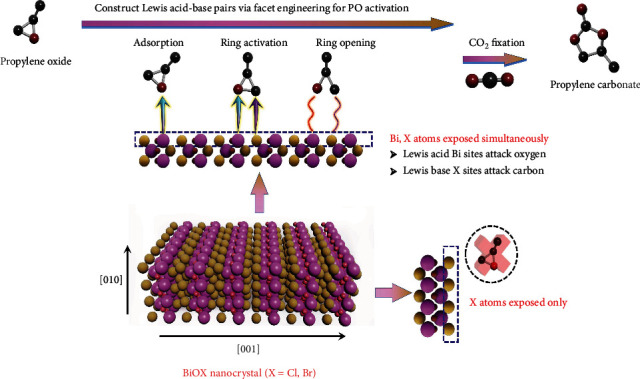
Illustration of CO_2_ cycloaddition with epoxides on different facets of BiOX.

Given that the epoxide ternary ring contains oxygen atom with lone pair of electrons, meanwhile carbon atoms are attackable by nucleophilic reagents, it is reasonable to construct Lewis acid-base pairs that can simultaneously act as electron acceptors and donors to activate epoxide. Further, Lewis acid-base pairs could also be beneficial to the adsorption and activation of CO_2_. Based on this, we proposed to construct Lewis acid-base pairs for effective ring-opening of epoxides, particularly with nonfree halide ions as Lewis base sites. Considering the unique confined layered structure of bismuth oxyhalides (BiOX, X = Cl, Br, I) with alternate positive [Bi_2_O_2_] layer and double negative halogen layers [25], the active surface can be obtained by appropriate design containing nonfree nucleophilic halide ions, which can cooperate with the Bi sites to form Lewis acid-base pairs to catalyze the CO_2_ cycloaddition of epoxides reaction. For example, bismuth oxybromide (BiOBr) holding the structure of [Bi_2_O_2_]^2+^ layers interleaving with double layers of bromine atoms becomes an ideal model for our study. As shown in Scheme 1, only Lewis base Br sites are exposed in BiOBr with (001) crystal facets. In comparison, Lewis acid Bi sites and Lewis base Br sites are exposed simultaneously for (010) crystal facets, where Bi atom can accept electrons from oxygen atoms in epoxides, and Br atom can provide electrons to carbon atoms in epoxide ternary rings. Thus, it is anticipated that effectively CO_2_ cycloaddition reaction could be accomplished by facet engineering on BiOBr nanocrystal to construct Lewis acid-base pairs.

## 2. Results and Discussion

**Figure 1:**
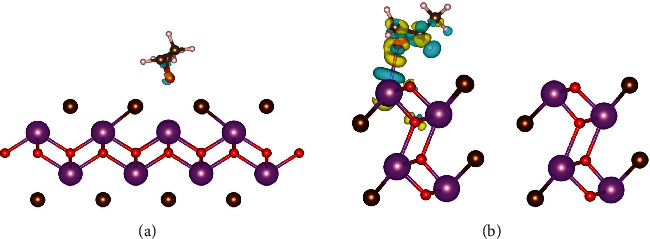
Theoretical study. Calculated deformation charge density of PO adsorbing on the surface of (a) BiOBr-(001) and (b) BiOBr-(010).

To get deeper insights into the role of Lewis acid-base pairs in epoxide and CO_2_ activation, density functional theory (DFT) was employed to investigate the adsorption behavior of propylene oxide (PO) and CO_2_ on different facets of BiOBr models, as shown in Figure 1 and S1. Compared to the slab of BiOBr-(001), the slab of BiOBr-(010) with the exposed Lewis acid-base sites exhibited more negative adsorption energy for both PO and CO_2_ (Table S2), indicating its good adsorption ability for reactants. Meanwhile, the Bader charge analysis revealed that almost no electron transfer occurred in the activation of PO and CO_2_ on the slab of BiOBr-(001) (Table S3), while the product propylene carbonate (PC) had barely any electron transfer on the slab of BiOBr-(010), indicating that the reaction was selective up to the PC, as well as facilitating the detachment of the product.

**Figure 2:**
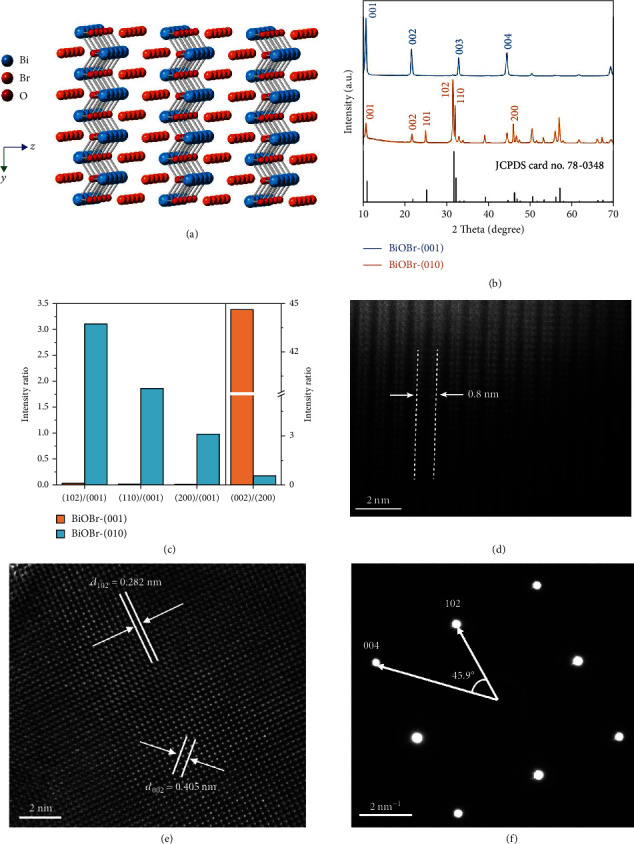
Structural characterization of BiOBr nanoplates. (a) atomic structure of (010) surfaces of the side view. (b) XRD patterns. (c) XRD peak intensity ratios of several key crystal facets for BiOBr-(001) and BiOBr-(010). (d) HAADF-STEM image of BiOBr-(010). (e) HRTEM and (f) SAED of BiOBr-(010).

In order to study this issue, herein the BiOBr nanoplates with exposed (001) and (010) crystal facets were obtained by a modified hydrothermal method, which donated as BiOBr-(001) and BiOBr-(010), respectively (See detail in Supporting Information). As a well characteristic method for determining the orientation of crystal growth, X-ray diffraction (XRD) patterns display that both BiOBr samples exhibiting peak positions could be indexed to the tetragonal phase BiOBr with high purity and no other impurity peaks (Figure 2(b)). To better compare the differences in XRD peak intensities of samples with different crystal facets, the intensity ratios of several key crystal facets were calculated (Figure 2(c)). [26] It is remarkable that the peak intensities of (00 L) facets in BiOBr-(001) sample are prominently higher than that of BiOBr-(010) sample. Furthermore, for BiOBr-(010), the intensity ratios of (102), (110), and (200) to (001) are distinctively higher except (002) to (200) than those of BiOBr-(001) sample, indicating that BiOBr-(001) and BiOBr-(010) have distinctly diverse growth directions. The full spectral sweep of the X-ray photoelectron spectroscopy (XPS) shows that both samples are free of impurity elements, further illustrating the high purity of the phases (Figure S2). Moreover, scanning electron microscopy (SEM) and transmission electron microscopy (TEM) images showed the plate-like morphology of the BiOBr samples, with a thickness of 40−60 nm (Figures S3 and S4). To further study the surface atomic structures of the BiOBr samples at atomic scale, the aberration-corrected high-angle annular dark-field scanning transmission electron microscopy (HAADF-STEM) image of BiOBr-(010) exhibits bright stripes with distance of ~0.8 nm, which can correspond to the atomic arrangement of bismuth element in [Bi_2_O_2_]^2+^ slabs (Figure 2(d)) [25]. The high-resolution TEM (HRTEM) image shows that BiOBr-(010) has a monocrystalline structure (Figure 2(e)), with the distances between adjacent lattice fringes measured as 2.82 Å and 4.05 Å which correspond to the (102) and (002) planes of BiOBr, respectively. In addition, the selective area electron diffraction (SAED) shows (102) and (004) planes, confirming the orientation of [010] and the high purity of the sample (Figure 2(f)) [27, 28]. For comparison, HAADF-STEM, HRTEM, and SAED of BiOBr-(001) confirmed the dominant exposed surfaces to be (001) facets (Figures S5 and S6). In summary, BiOBr nanoplates with different exposed facets were successfully prepared.

Due to the surface atomic structure and coordination environment of the catalyst directly determining its properties, the surface structure of the (001) and (010) facets of BiOBr were then investigated. The (001) facet shows a closed structure consisting of the most outer layer of Br atoms, whereas the (010) facet has alternating Bi, O, and Br atoms at the outermost layer (Figure 2(a) and S7) [26]. Thus, the Lewis acid-base pairs can be constructed simultaneously by controlling the exposure of the (010) facets of the BiOBr nanoplates.

**Figure 3:**
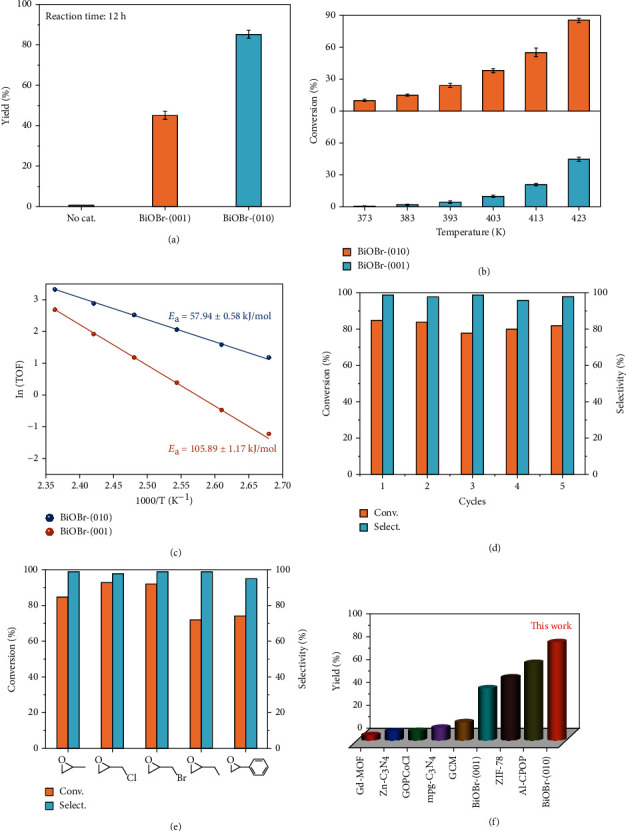
Catalytic tests. (a) Performances of BiOBr samples for CO_2_ cycloaddition with PO. Reaction condition: 8 bar CO_2_ at 423 K. (b) Conversion for BiOBr-(001) and BiOBr-(010) at different temperatures. (c) The Arrhenius plots of BiOBr-(001) and BiOBr-(010). (d) Stability cyclic test for BiOBr-(010). Reaction time for each cycle: 12 h. (e) Catalytic performance of BiOBr-(010) toward the CO_2_ cycloaddition with different epoxides. (f) Catalytic performance of various catalysts toward the CO_2_ cycloaddition with epoxides reaction.

With abundant Lewis acid-base pairs on the surface, BiOBr-(010) would exhibit excellent PO activation ability, which facilitates efficient catalytic CO_2_ cycloaddition reaction. To investigate the role of Lewis acid-base pairs in CO_2_ cycloaddition, BiOBr-(001) and BiOBr-(010) nanoplates were employed for the synthesis of PC from CO_2_ and PO. The cycloaddition reaction of carbon dioxide with PO was carried out in a closed pressurized reactor under 8 bar CO_2_ at 423 K. As shown in Figure 3(a), it is a surprise that the conversion yield of BiOBr-(010) with rich Lewis acid-base pairs achieved up to 85% under cocatalyst-free condition, which was ~1.8-fold higher than that of BiOBr-(001), suggesting that Lewis acid-base pairs play a positive effect in the CO_2_ cycloaddition reaction. To further study the differences in the catalytic property of the samples, a series of catalytic reactions were tested at different temperatures while maintaining other factors unchanged. It is clearly seen from Figure 3(b) that BiOBr-(010) nanoplates shows a greater catalytic reactivity with higher conversion at all temperature conditions. The TOF number was calculated by controlling the reaction time to obtain the conversion of the reaction at the initial stage for a more accurate comparison of the catalytic activity. Moreover, the activation energy (*E*_a_) values were calculated based on the Arrhenius plots, which were obtained by linearly fitting the curve of ln(TOF) to 1000/T. As shown in Figure 3(c), BiOBr-(010) exhibited a much lower activation energy (*E*_a_ = 57.94 ± 0.58 kJ/mol), which was only about half of that for BiOBr-(001) (*E*_a_ = 105.89 ± 1.17 kJ/mol). Therefore, BiOBr-(010) can lower the activation energy of the reaction more effectively and greatly enhance the catalytic activity without cocatalyst. As mentioned above, BiOBr-(010) possesses a higher conversion rate and a lower activation energy of the reaction under the same reaction conditions.

In addition, to verify the robust stability of the sample during the reaction, a catalytic cycle stability test was performed on BiOBr-(010). After five catalytic cycles, BiOBr-(010) still maintained its original catalytic activity with no decrease in conversion and selectivity (Figure 3(d)). In addition, the XRD and TEM after the reaction displayed that the original morphology and structure of BiOBr-(010) was still maintained (Figure S12). Besides, the scope of BiOBr-(010) catalyst in other epoxy-based substrates was examined to further evaluate its substrate tolerance and versatility. As shown in Figure 3(e) and Table S1, epichlorohydrin, epibromohydrin, 1,2-epoxybutane, and styrene could be effectively transformed into corresponding cyclic organic carbonates under cocatalyst-free condition, excepting for cyclohexene oxide. The relatively low conversion of cyclohexene oxide was attributed to the steric effect, which blocked the interaction of the epoxide with the active site on the catalyst surface. More importantly, the conversion and selectivity for BiOBr-(010) significantly surpassed previously reported catalysts (Figure 3(f) and Table S4). [22, 29] Therefore, BiOBr-(010) with abundant Lewis acid-base pairs were efficient catalysts for the CO_2_ cycloaddition with epoxides in lack of cocatalysts. The same facet engineering method is also work for BiOCl samples, where the conversion of PO via BiOCl-(010) is up to 86%, ~1.9 times greater than that of BiOCl-(001) (Figure S17).

**Figure 4:**
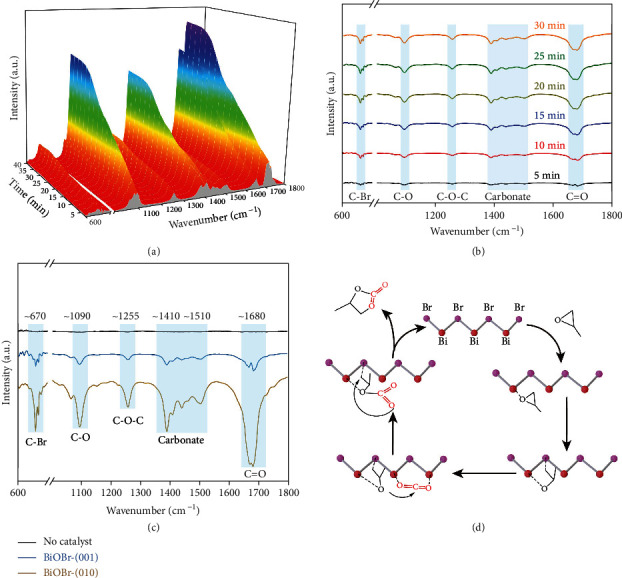
Reaction mechanism study. (a) *In-situ* DRIFTS spectra for CO_2_ cycloaddition with PO for BiOBr-(010). (b) 2D plot of *in-situ* DRIFTS spectra for CO_2_ cycloaddition with PO for BiOBr-(010). (c) *In-situ* DRIFTS spectra for the catalysts with CO_2_ and PO for 30 min. d) Proposed mechanism for the CO_2_ cycloaddition with PO catalyzed by BiOBr-(010).

With the enrichment of Lewis acid-base pairs on the surface, BiOBr-(010) exhibits outstanding catalytic performance in CO_2_ cycloaddition reaction, and it is essential to investigate the mechanism of the reaction to gain a clearer and more definite understanding of the overall catalytic reaction. To further study the crucial role of Lewis acid-base pairs in the ring-opening of PO and the CO_2_ cycloaddition reaction, *in-situ* diffuse reflectance infrared Fourier-transform spectroscopy (DRIFT) was performed to study the reaction intermediates by observing bonding alterations in the catalytic reaction. As shown in Figure 4(a), after 30 min treatment with PO under a carbon dioxide atmosphere at 423 K, five new peaks at 670, 1090, 1255, 1410, 1510, and 1680 cm^−1^ appeared, corresponding to the vibration of C-Br, C-O, C-O-C, carbonate, and C = O, respectively [30–34]. Of note, the formation of C-Br demonstrated that Br atoms acted as a Lewis base sites to attack the carbon atoms in the epoxide, which played a key role in the ring-opening of the epoxide. The intensity of each peak strengthened as time increased. Meanwhile, comparing the signals collected at 30 min for both samples, the all above peaks of BiOBr-(010) nanoplates were significantly stronger than that of BiOBr-(001), suggesting the high catalytic activity of BiOBr-(010). More importantly, despite BiOBr-(010) had lower density Br sites compared to BiOBr-(001), the C-Br peak of BiOBr-(010) was much higher than that of BiOBr-(001), which forcefully confirmed Lewis acid-base pairs in (010) facet of BiOBr were efficient for the activation of epoxide.

Further, DFT calculations were conducted to illustrate the origin of catalytic activity on BiOBr models with different facets. As seen from the calculated density of states (DOS), compared to (001) facet of BiOBr, the disappearance of the O peak near the Fermi energy level in the DOS of (010) facet is accompanied by a decrease in the area of the nearest neighbouring Bi nonbonded peak, which is caused by the electron injection into Bi from the O of PO (Figure S15). Furthermore, combined with the Bader charge analysis (Table S3), it was found that PO lost 0.04 electrons on (010) facet of BiOBr, while almost no electrons were transferred on (001) facet, suggesting that the Bi sites on (010) facet act as Lewis acid sites. In other words, the simultaneous presence of the Bi and Br sites on (010) facet of BiOBr achieved the construction of Lewis acid-base pairs. Considering the unique ternary ring structure of PO, the first step in the activation of PO is actually the electron transfer from O atom of PO to Bi site, which weakens the epoxide bond, and then Br site attacks C atom as a nucleophilic reagent (Lewis base), so that effective ring-opening can take place under relatively mild conditions. It is worth mentioning that the adsorption of CO_2_ on the (010) facet of BiOBr is easier than that of (001) owing to the existence of Lewis acid-base sites (Figure S9). Subsequently, the oxygen in the activated ring-opened epoxide interacts with CO_2_ to form a carbonate structural intermediate, which is ultimately converted to the corresponding cyclic carbonate *via* a ring-closing step (Figure 4(d)). The above analysis further explains the predominance of constructing Lewis acid-base pairs in PO activation and CO_2_ cycloaddition reaction. In contrast, for the (001) facet of BiOBr with only the Lewis base Br sites, more harsh reaction conditions would be required. Benefitting from the synergy of the dual active sites, BiOBr-(010) can effectively catalyze the CO_2_ cycloaddition with epoxides in a moderate and cocatalyst-free condition.

## 3. Conclusion

In summary, a facet engineering strategy was proposed to construct Lewis acid-base pairs for CO_2_ cycloaddition, where BiOX (X = Cl, Br) with exposed specific facet could effectively activate CO_2_ and PO in producing high value-added cycle carbonates. By taking BiOBr nanoplates as a model system, both theoretical calculations and experiments demonstrated that BiOBr-(010) with Lewis acid-base pairs has a stronger CO_2_ and PO activation capacity in comparison with BiOBr-(001). In detail, the synergistic interaction between the Lewis acid Bi sites and the Lewis base Br sites coexisted in BiOBr-(010) promotes the ring opening of the epoxide as well as the efficient activation of CO_2_ without any cocatalyst as evidenced by *in-situ* DRIFT. To go further, the mechanism investigation based on Bader charge calculations and DOS evidenced that the electrons of O atom in PO was transferred to Lewis acid Bi sites, accompanied by the attack of Lewis base Br sites to C atom of PO, leading to effective ring-opening under relatively mild conditions. Benefiting from the simultaneous exposure of Lewis acid Bi sites and the Lewis base Br sites, BiOBr-(010) showed a high conversion yield of 85% with almost 100% selectivity in the catalytic CO_2_ cycloaddition reaction with epoxides without cocatalyst. This work not only provides insight into the design of a cocatalyst-free catalyst in CO_2_ cycloaddition, but also gives a deep understanding of the mechanism of CO_2_ cycloaddition reactions.

## 4. Materials and Methods

Samples synthesis and additional characterization are included in the Supplementary Materials (available here).

## Data Availability

All data needed to evaluate the conclusions in the paper are presented in the paper and/or the Supplementary Materials. Additional data related to this paper may be requested from the authors.

## Supplementary Materials

Supplementary Materials Figure S1. CO_2_ absorption on the surface of (a) BiOBr-(001) and (b) BiOBr-(010). Figure S2. The components and chemical state revealed by the XPS survey scan spectrum of BiOBr-(001) and BiOBr-(010) samples, revealing the purity of the samples. Figure S3. SEM image of as-synthesized (a) BiOBr-(001) and (b) BiOBr-(010) samples. Figure S4. TEM image of (a) BiOBr-(001) and (b) BiOBr-(010) sample. Figure S5. HRTEM and SAED of BiOBr-(001) nanoplates. Figure S6. HAADF-STEM image of BiOBr-(001). Figure S7. Atomic structure of BiOBr-(001) surfaces of the side view. Figure S8. N_2_ adsorption-desorption isotherm of (a) BiOBr-(001) and (b) BiOBr-(010). Figure S9. CO_2_ adsorption isotherms of BiOBr samples. Figure S10. Representative ^1^H-NMR spectra of the products for Cycloaddition of Epoxides with CO_2_ in dimethylformamide (DMF) solution after 12 h reaction. Figure S11. In-situ DRIFTS spectra for CO_2_ cycloaddition with PO with TBAB for 30 min. Figure S12. Characterization of BiOBr-(010) sample after 5 catalytic cycles. (a) XRD pattern and (b) TEM image after catalytic cycles up to 60 hours. Figure S13. The XPS spectra of Bi 4f for BiOBr-(010) before and after catalysis. Figure S14. Detection experiments for leaching of Br ions. Figure S15. Calculated density of states of PO adsorbed on (a). (001)- and (b). (010)- facet of BiOBr. Figure S16. XRD patterns of the BiOCl samples. Figure S17. Performances of BiOCl samples for CO_2_ cycloaddition with PO. Reaction condition: CO_2_ pressure of 8 bar at 423 K. Figure S18. HRTEM, SAED, and corresponding EDS mapping images of BiOCl-(001) (a-c) and BiOCl-(010) (d-f). Table S1. Cycloaddition of Epoxides with CO_2_. Table S2. Absorption energy of CO_2_, PO and PC on (001) and (010) facets of BiOBr. Table S3. Bader charge analysis of CO_2_, PO and PC on (001) and (010) facets of BiOBr. Table S4. Cycloaddition of CO_2_ and PO using different catalysts under various reaction conditions without addition of co-catalyst. Table S5. Comparison of performances of traditional metal oxides for cycloaddition of CO_2_ and PO.

## References

[1] Bushuyev O. S., De Luna P., Dinh C. T. (2018). What should we make with CO_2_ and how can we make it?. *Joule*.

[2] Yang Q., Peng H., Zhang Q. (2021). Atomically dispersed high-density Al–N4Sites in porous carbon for efficient photodriven CO_2_ cycloaddition. *Advanced Materials*.

[3] Zhang X., Sa R., Zhou F. (2021). Metal–organic framework-derived CuS nanocages for selective CO_2_ electroreduction to formate. *CCS Chem.*.

[4] Gu J., Hsu C.-S., Bai L., Chen H. M., Hu X. (2019). Atomically dispersed Fe^3+^sites catalyze efficient CO_2_ electroreduction to CO. *Science*.

[5] Huang H., Ye J.-H., Zhu L. (2021). Visible-light-driven anti-markovnikov hydrocarboxylation of acrylates and styrenes with CO_2_. *CCS Chem.*.

[6] Liu Q., Wu L., Jackstell R., Beller M. (2015). Using carbon dioxide as a building block in organic synthesis. *Nature Communications*.

[7] Masuda Y., Ishida N., Murakami M. (2015). Light-driven carboxylation of o-alkylphenyl ketones with CO_2_. *Journal of the American Chemical Society*.

[8] Jin S., Shao W., Chen S. (2022). Ultrathin in-plane heterostructures for efficient CO_2_ chemical fixation. *Angewandte Chemie, International Edition*.

[9] Zhang X., Liu H., An P. (2020). Delocalized electron effect on single metal sites in ultrathin conjugated microporous polymer nanosheets for boosting CO_2_ cycloaddition. *Science Advances*.

[10] de la Cruz-Martínez F., Martínez J., Gaona M. A. (2018). Bifunctional aluminum catalysts for the chemical fixation of carbon dioxide into cyclic carbonates. *ACS Sustain. Chem. Amp Eng.*.

[11] Scharfenberg M., Hilf J., Frey H. (2018). Functional polycarbonates from carbon dioxide and tailored epoxide monomers: degradable materials and their application potential. *Advanced Functional Materials*.

[12] Luinstra G. (2008). Poly(propylene carbonate), Old Copolymers of Propylene Oxide and Carbon Dioxide with New Interests: Catalysis and Material Properties. *Polymer Reviews*.

[13] Matsukizono H., Endo T. (2018). Reworkable Polyhydroxyurethane films with reversible acetal networks obtained from multifunctional six-membered cyclic carbonates. *Journal of the American Chemical Society*.

[14] Baral E. R., Lee J. H., Kim J. G. (2018). Diphenyl carbonate: a highly reactive and green carbonyl source for the synthesis of cyclic carbonates. *The Journal of Organic Chemistry*.

[15] Liu M., Li X., Liang L., Sun J. (2016). Protonated triethanolamine as multi-hydrogen bond donors catalyst for efficient cycloaddition of CO_2_ to epoxides under mild and cocatalyst-free conditions. *Journal of CO₂ Utilization*.

[16] Hui W., He X.-M., Xu X.-Y. (2020). Highly efficient cycloaddition of diluted and waste CO_2_ into cyclic carbonates catalyzed by porous ionic copolymers. *Journal of CO₂ Utilization*.

[17] Byun J., Zhang K. A. I. (2018). Controllable homogeneity/heterogeneity switch of imidazolium ionic liquids for CO_2_ utilization. *ChemCatChem*.

[18] Chen S., Pudukudy M., Yue Z. (2019). Nonmetal Schiff-base complex-anchored cellulose as a novel and reusable catalyst for the solvent-free ring-opening addition of CO_2_ with epoxides. *Ind. Amp Eng. Chem. Res.*.

[19] Natongchai W., Posada-Pérez S., Phungpanya C. (2022). Enhancing the catalytic performance of group I, II metal halides in the cycloaddition of CO_2_ to epoxides under atmospheric conditions by cooperation with homogeneous and heterogeneous highly nucleophilic aminopyridines: experimental and theoretical study. *The Journal of Organic Chemistry*.

[20] Xiong J., Li J., Chen C. (2020). Crosslinked resin-supported bifunctional organocatalyst for conversion of CO_2_ into cyclic carbonates. *ChemSusChem*.

[21] Cole-Hamilton D. J. (2003). Homogeneous catalysis--new approaches to catalyst separation, recovery, and recycling. *Science*.

[22] Xue Z., Jiang J., Ma M.-G., Li M.-F., Mu T. (2017). Gadolinium-based metal–organic framework as an efficient and heterogeneous catalyst to activate epoxides for cycloaddition of CO_2_ and alcoholysis. *ACS Sustain. Chem. Amp Eng.*.

[23] Li Y., Song X., Zhang G. (2022). Cobalt sandwich complex-based covalent organic frameworks for chemical fixation of CO_2_. *Science China Materials*.

[24] Wu X., Chen C., Guo Z., North M., Whitwood A. C. (2019). Metal- and halide-free catalyst for the synthesis of cyclic carbonates from epoxides and carbon dioxide. *ACS Catalysis*.

[25] Wang H., Chen S., Yong D. (2017). Giant electron–hole interactions in confined layered structures for molecular oxygen activation. *Journal of the American Chemical Society*.

[26] Shi M., Li G., Li J. (2020). Intrinsic facet-dependent reactivity of well-defined BiOBr nanosheets on photocatalytic water splitting. *Angewandte Chemie, International Edition*.

[27] Xi Y., Chen W., Dong W. (2021). Engineering an interfacial facet of S-scheme heterojunction for improved photocatalytic hydrogen evolution by modulating the internal electric field. *ACS Applied Materials & Interfaces*.

[28] Wu X., Ng Y. H., Wang L. (2017). Improving the photo-oxidative capability of BiOBr via crystal facet engineering. *Journal of Materials Chemistry A*.

[29] Liu J., Wang A., Jing H. (2020). Chemical fixation of carbon dioxide catalyzed via hydroxyl and carboxyl-rich glucose carbonaceous material as a heterogeneous catalyst. *Chemical Engineering Journal*.

[30] Zhang J., Sato H., Tsuji H. (2020). Debromination and decomposition mechanisms of phenolic resin molecules in ball milling with nano-zerovalent iron. *ACS Sustain. Chem. Amp Eng.*.

[31] Zhang J., Sato H., Tsuji H., Noda I., Ozaki Y. (2005). Infrared spectroscopic study of CH3···OC interaction during poly(L-lactide)/poly(D-lactide) stereocomplex formation. *Macromolecules*.

[32] Moradzaman M., Mul G. (2020). Infrared analysis of interfacial phenomena during electrochemical reduction of CO_2_ over polycrystalline copper electrodes. *ACS Catalysis*.

[33] Baruch M. F., Pander J. E., White J. L., Bocarsly A. B. (2015). Mechanistic insights into the reduction of CO_2_ on tin electrodes using in situ ATR-IR spectroscopy. *ACS Catalysis*.

[34] Yan L., Hou L., Sun S., Wu P. (2020). Dynamic diffusion of disperse dye in a polyethylene terephthalate film from an infrared spectroscopic perspective. *Ind. Amp Eng. Chem. Res.*.

