# Supplementary material for: Facet Engineering in Constructing Lewis Acid-Base Pairs for CO2 Cycloaddition to High Value-Added Carbonates
Source: Research (Wash D C). 2022 Oct 14;2022:9878054. doi: 10.34133/2022/9878054 (PMC9590269; doi:10.34133/2022/9878054)
Supplement: Supplementary Materials — Figure S1. CO2 absorption on the surface of (a) BiOBr-(001) and (b) BiOBr-(010). Figure S2. The components and chemical state revealed by the XPS survey scan spectrum of BiOBr-(001) and BiOBr-(010) samples, revealing the purity of the samples. Figure S3. SEM image of as-synthesized (a) BiOBr-(001) and (b) BiOBr-(010) samples. Figure S4. TEM image of (a) BiOBr-(001) and (b) BiOBr-(010) sample. Figure S5. HRTEM and SAED of BiOBr-(001) nanoplates. Figure S6. HAADF-STEM image of BiOBr-(001). Figure S7. Atomic structure of BiOBr-(001) surfaces of the side view. Figure S8. N2 adsorption-desorption isotherm of (a) BiOBr-(001) and (b) BiOBr-(010). Figure S9. CO2 adsorption isotherms of BiOBr samples. Figure S10. Representative 1H-NMR spectra of the products for Cycloaddition of Epoxides with CO2 in dimethylformamide (DMF) solution after 12 h reaction. Figure S11. In-situ DRIFTS spectra for CO2 cycloaddition with PO with TBAB for 30 min. Figure S12. Characterization of BiOBr-(010) sample after 5 catalytic cycles. (a) XRD pattern and (b) TEM image after catalytic cycles up to 60 hours. Figure S13. The XPS spectra of Bi 4f for BiOBr-(010) before and after catalysis. Figure S14. Detection experiments for leaching of Br ions. Figure S15. Calculated density of states of PO adsorbed on (a). (001)- and (b). (010)- facet of BiOBr. Figure S16. XRD patterns of the BiOCl samples. Figure S17. Performances of BiOCl samples for CO2 cycloaddition with PO. Reaction condition: CO2 pressure of 8 bar at 423 K. Figure S18. HRTEM, SAED, and corresponding EDS mapping images of BiOCl-(001) (a-c) and BiOCl-(010) (d-f). Table S1. Cycloaddition of Epoxides with CO2. Table S2. Absorption energy of CO2, PO and PC on (001) and (010) facets of BiOBr. Table S3. Bader charge analysis of CO2, PO and PC on (001) and (010) facets of BiOBr. Table S4. Cycloaddition of CO2 and PO using different catalysts under various reaction conditions without addition of co-catalyst. Table S5. Comparison of performances of t [file 9878054.f1.docx]

Supplementary Materials

**Facet Engineering in Constructing Lewis Acid-base Pairs for CO_2_ Cycloaddition to high value-added** **Carbonates**

Shu Shang^1#^, Wei Shao^1#^, Xiao Luo^1^, Ming Zuo^1^, Hui Wang^1^, Xiaodong Zhang^1,2,^*, and Yi Xie^1,2,^*

^1^ Hefei National Research Center for Physical Sciences at the Microscale, University of Science and Technology of China, Hefei 230026, China

^2^ Institute of Energy, Hefei Comprehensive National Science Center, Hefei 230031, China

^#^ These authors contributed equally to this work.

Correspondence should be addressed to Xiaodong Zhang; [zhxid@ustc.edu.cn](mailto:zhxid@ustc.edu.cn) and Yi Xie; [yxie@ustc.edu.cn](mailto:yxie@ustc.edu.cn)

**Experimental Procedures**

**Materials**

Bismuth(III) nitrate pentahydrate (Bi(NO_3_)_3_∙5H_2_O), Potassium bromide (KBr), Sodium hydroxide (NaOH), N, N-Dimethylformamide (DMF), Tetrabutylammonium bromide (TBAB) were purchased from Sinopharm Chemical Reagent Co., Ltd. Hexadecyl trimethyl ammonium Bromide (CTAB) and Hexadecyl trimethyl ammonium Chloride (CTAC) were purchased from Aladdin. The water used in all experiments was de-ionized (DI). All chemicals were used as received without further purification.

**Preparation of samples**

Preparation of BiOX-(010): Typically, 2 mmol Bi(NO_3_)_3_∙5H_2_O and 2 mmol of KX (X=Cl, Br) were successively added into 30 mL distilled water and stirred continuously for 60 min. Then the pH value of solution was adjusted to 6.0 with 2 M NaOH solution. The mixture solution was poured into a 50 mL Teflon lined stainless autoclave and maintained at 160℃ for 24 h. After naturally cooled to room temperature, the precipitate was collected and washed with distilled water and ethanol each for three times and then dried in vacuum to obtain BiOX-(010) sample.(*1*)

Preparation of BiOX-(001): Typically, 600 mg Bi(NO_3_)_3_∙5H_2_O and 600 mg of CTAB (for BiOBr) or CTAC (for BiOCl) were dissolved in 30 mL deionized water by stirring for 1 h, and then transferred into 50 mL Teflon lined stainless autoclave and maintained at 160℃ for 18 h. After naturally cooled to room temperature, the precipitate was collected and washed with distilled water and ethanol each for three times and then dried in vacuum to obtain BiOX-(001) sample.(*2*)

**Characterization**

Powder X-ray diffraction patterns (PXRD) were carried out on a Japan Rigaku MiniFlex 600 equipped with graphite-monochromated Cu Kα radiation (λ = 1.54178 Å). Scanning electron microscopy (SEM) images were taken on a FEI Sirion-200 field emission scanning electron microscope operated at 5 kV. Transmission electron microscopy (TEM) images were recorded on a Hitachi-H7650 transmission electron microscope. High-resolution transmission electron microscopy (HRTEM) and corresponding electron diffraction (ED) analyses were carried out on a JEOL JEM-ARF200F. X-ray photoelectron spectra (XPS) were acquired on a Thermo ESCALAB 250 spectrometer with a Al Kα excitation source (h*ν* = 1486.6 eV). The binding energies derived from the XPS analysis were corrected against the specimen charging by referencing C 1s to 284.8 eV.

**Catalytic tests**

Typically, the Cycloaddition of Epoxides with CO_2_ reaction was carried out in a stainless-steel autoclave. After the addition of 13.5 ml of DMF, 4.5 mL epoxides and 50 mg catalysts into a Teflon inlet, the autoclave (NSVP100-P3-T2-HC1-SV-R, Anhui Kemi Machinery Technology Co., Ltd) was pressurized with high-purity CO_2_ pressure (99.99%, 0.8 MPa). The reaction was performed at 423 K with stirring at 800 r.p.m for 12 h. The liquid products were detected by NMR (400 MHz Bruker AVANCE AV III) spectroscopy.

***In-Situ* DRIFTS measurements**

*In-situ* diffuse reflectance infrared Fourier-transform spectroscopy (DRIFTS) measurements were obtained by using a Bruker IFS 66v Fourier-transform spectrometer equipped with a Harrick diffuse reflectance accessory at the Infrared Spectroscopy and Microspectroscopy Endstation (BL01B) in National Synchrotron Radiation Laboratory (NSRL) in Hefei, China. The samples were held in a custom-fabricated IR reaction chamber which was specifically designed to examine highly scattering powder samples in the diffuse reflection mode. The chamber was sealed with two ZnSe windows. During the *in-situ* characterization, 0.4 MPa of CO_2_ was introduced into the chamber and the peaks of free molecular CO_2_ were set as a reference between the samples. Each spectrum was recorded by averaging 128 scans at a 4 cm^-1^ spectral resolution.

**Calculation method**

DFT calculations were performed by using the Vienna Ab-initio Simulation Package (VASP) with the projector augmented wave method for the core region and a plane-wave kinetic energy cutoff of 480 eV. The generalized gradient approximation method with Perdew-Burke-Ernzerh of (PBE) functional for the exchange-correlation term was employed. The convergence of energy and forces were set to be less than 1×10^-5^ eV and 0.02 eV/Å, respectively. A set of 3×3×1 k-points were sampled in geometry optimizations and calculation of DOS (Density of States).

After geometry optimizations, adsorption energy was calculated as follows:

$$E_{ads}=E_{species@base}-E_{species}-E_{base}$$

where $E_{species@base}$ represent the energy of the adsorption configuration with different species as CO_2_, PO and PC. $E_{species}$ represent the energy of these species, $E_{base}$ represent the energy of different crystallographic planes as [001] and [010] of BiOBr structures after geometry in the previous step that can considered as ground state structure.


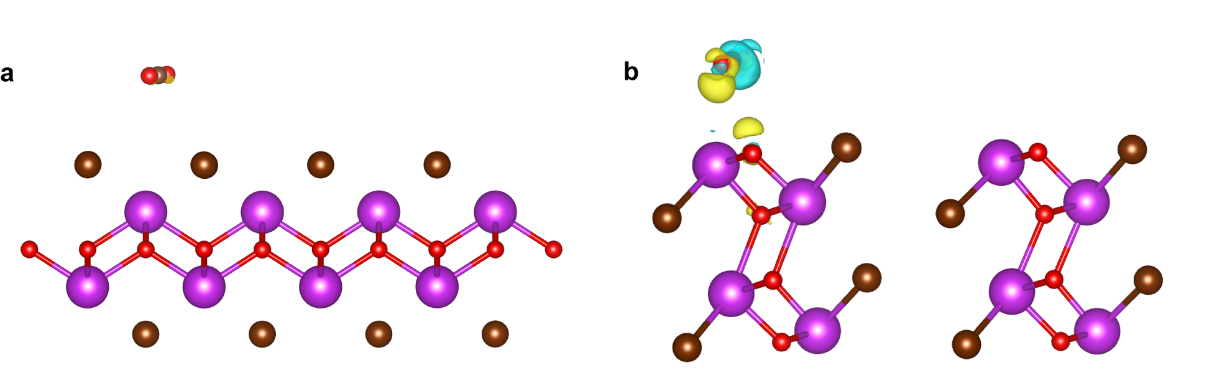


**Figure S1*.*** CO_2_ absorption on the surface of a) BiOBr-(001) and b) BiOBr-(010).


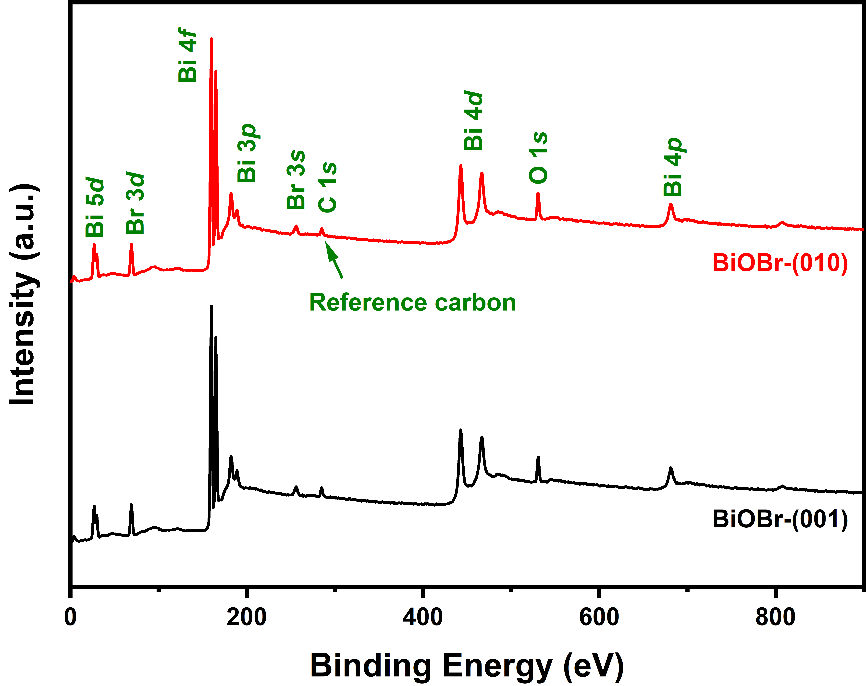


**Figure S2.** The components and chemical state revealed by the XPS survey scan spectrum of BiOBr-(001) and BiOBr-(010) samples, revealing the purity of the samples.


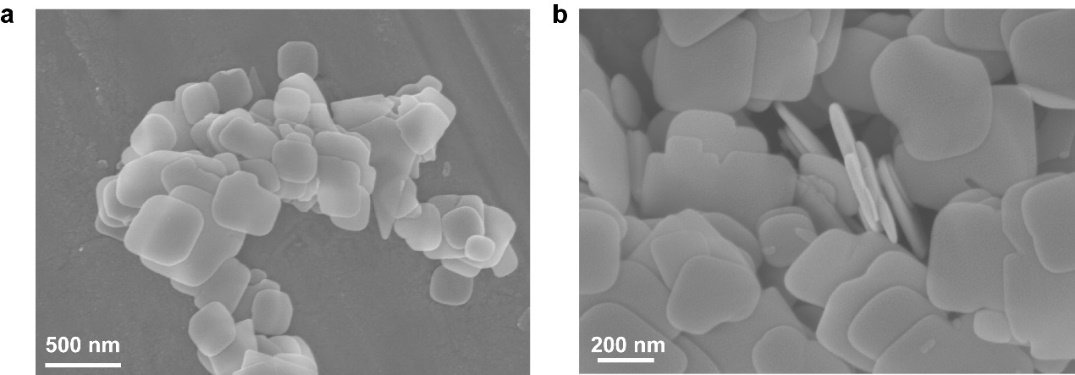


**Figure S3.** SEM image of as-synthesized (a) BiOBr-(001) and (b) BiOBr-(010) samples.


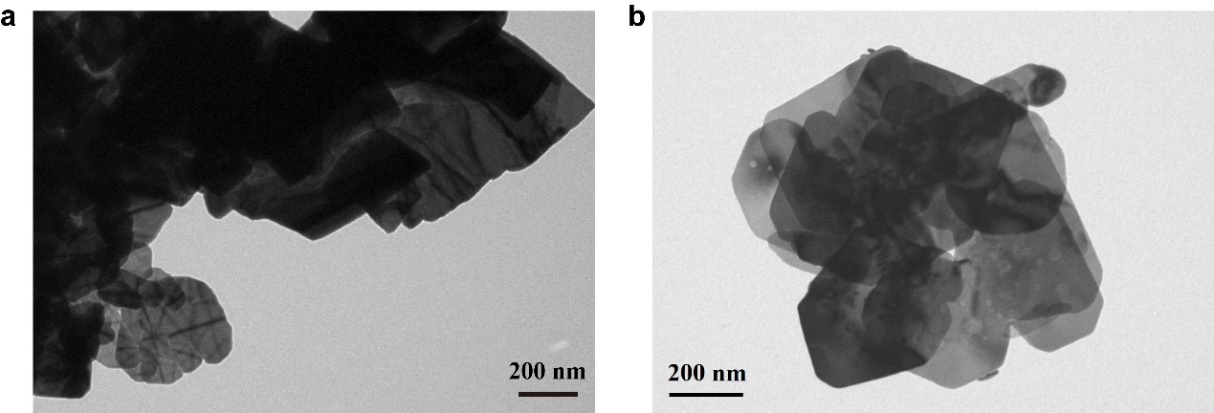


**Figure S4.** TEM image of (a) BiOBr-(001), (b) BiOBr-(010) sample.


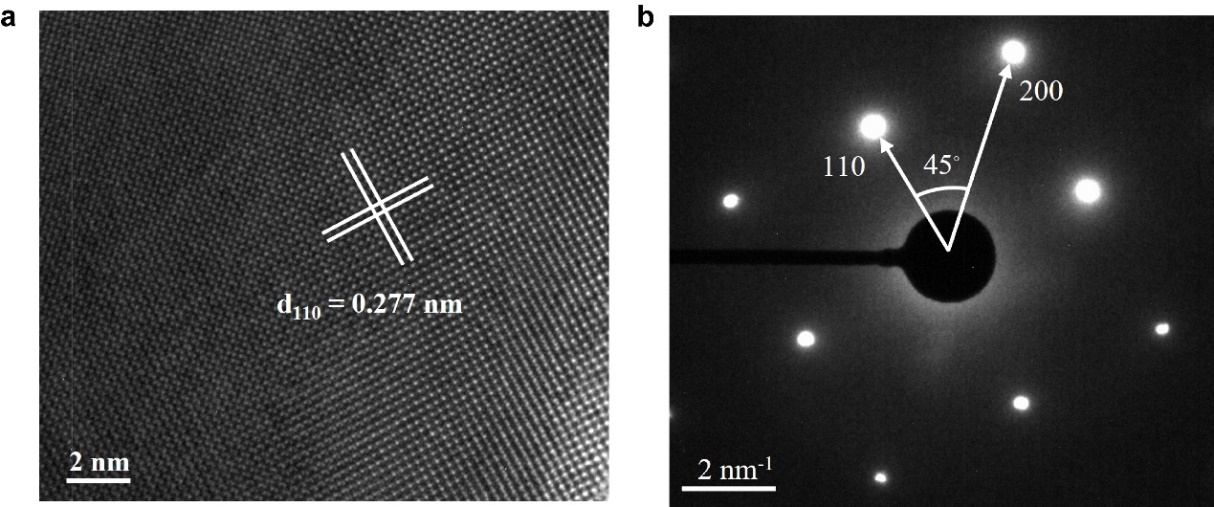


**Figure S5.** HRTEM and SAED of BiOBr-(001) nanoplates.


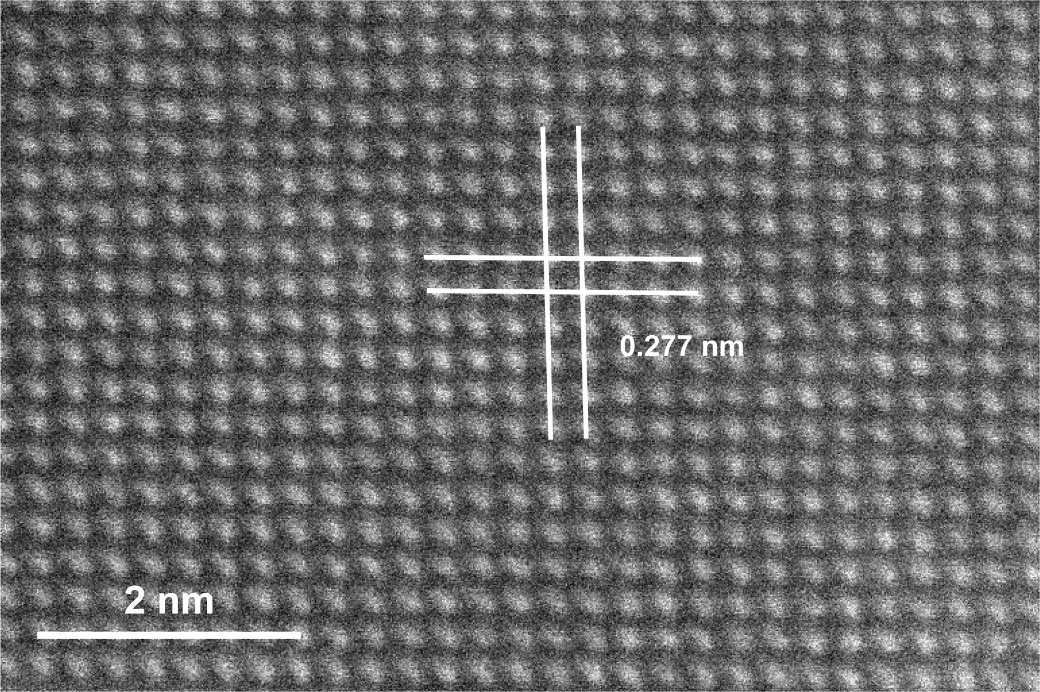


**Figure S6.** HAADF-STEM image of BiOBr-(001). The HAADF-STEM image of BiOBr-(001) exhibits interplanar distance of ~2.77 Å, which is well matched with the plane distances of d_110_, suggesting [001] orientation of the sample.


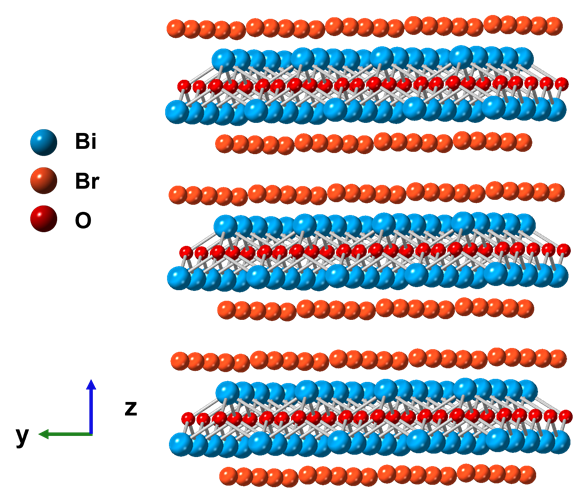


**Figure S7.** Atomic structure of BiOBr-(001) surfaces of the side view.


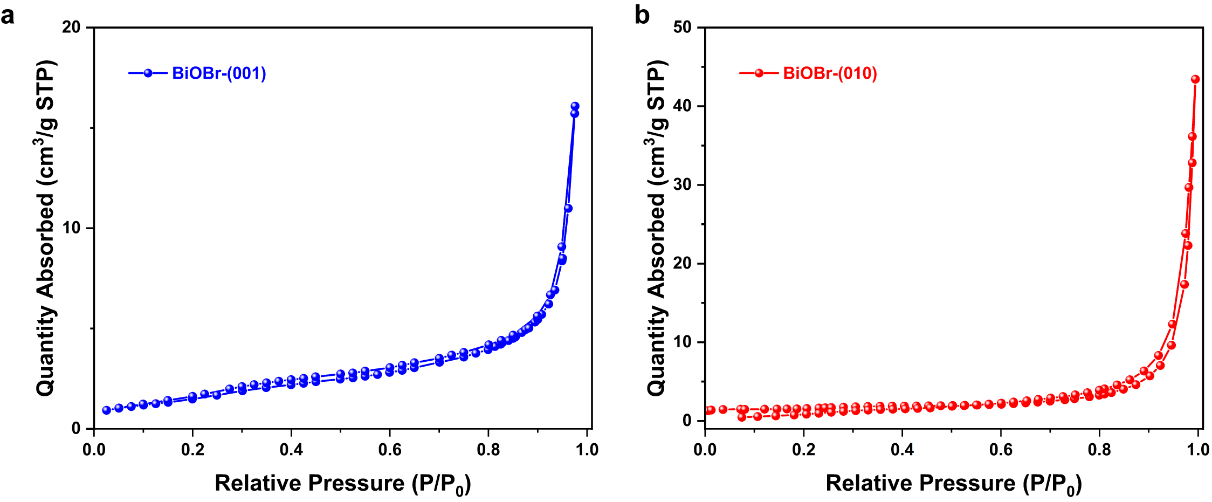


**Figure S8.** N_2_ adsorption-desorption isotherm of (a) BiOBr-(001) and (b) BiOBr-(010). The BET surface areas of BiOBr-(001) and BiOBr-(010) were 6.11 m^2^/g and 5.28 m^2^/g, respectively.


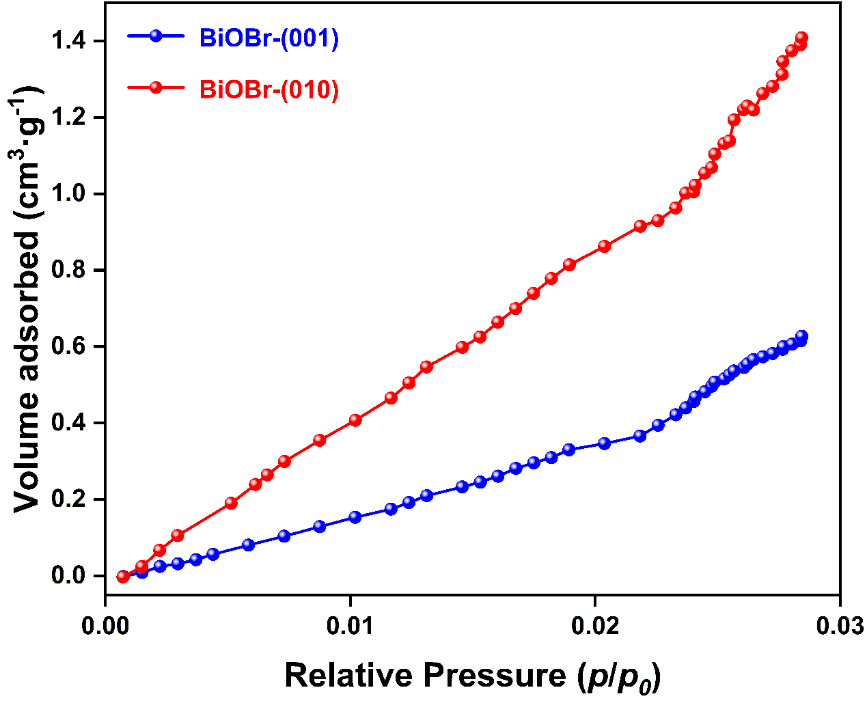


**Figure S9.** CO_2_ adsorption isotherms of BiOBr samples.


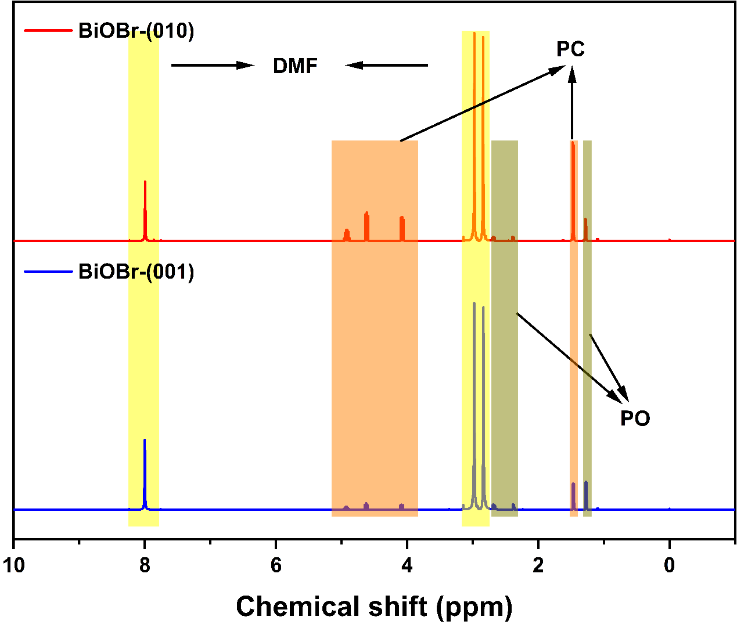


**Figure S10.** Representative ^1^H-NMR spectra of the products for Cycloaddition of Epoxides with CO_2_ in dimethylformamide (DMF) solution after 12 h reaction.


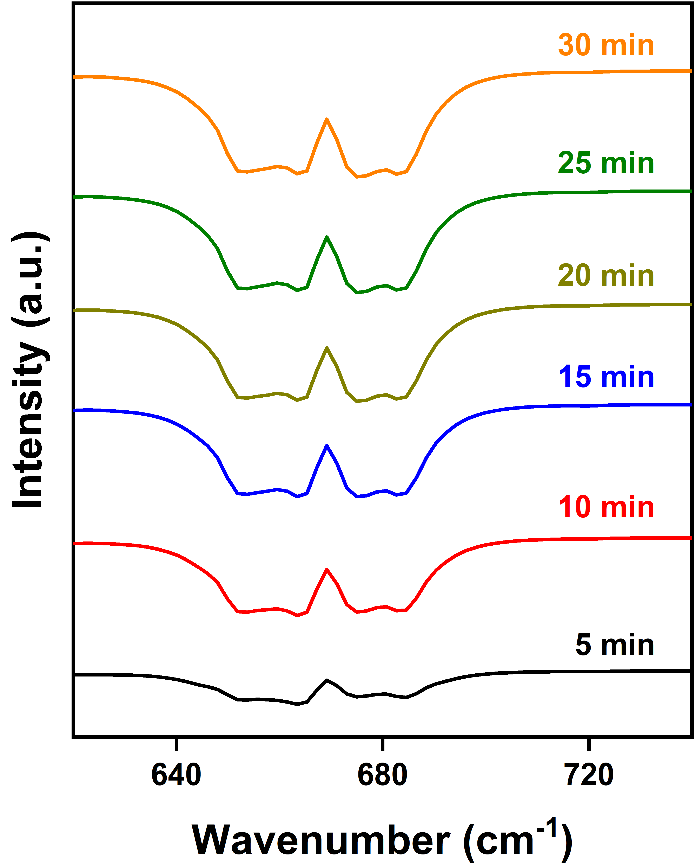


**Figure S11.** *In-situ* DRIFTS spectra for CO_2_ cycloaddition with PO with TBAB for 30 min. To further confirm that the peak ~670 cm^-1^ is attributed to the formation of C-Br bonds, we monitored the intermediates of the CO_2_ cycloaddition reaction with PO using TBAB as a catalyst by the DRIFT. As shown in Figure S11, peaks that increase in intensity with time were also observed ~670 cm^-1^, the shape and position of the peaks are consistent with those observed in the BiOBr catalytic system, and therefore we can provide sufficient evidence that the peaks at ~670 cm^-1^ can be attributed to C-Br bonds.


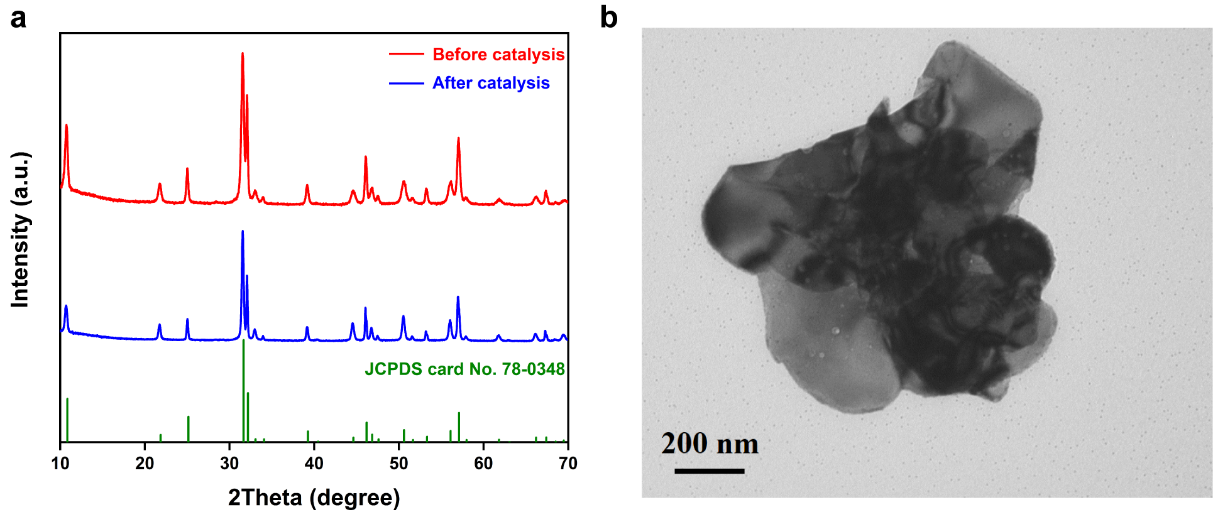


**Figure S12.** Characterization of BiOBr-(010) sample after 5 catalytic cycles. (a) XRD pattern, (b) TEM image after catalytic cycles up to 60 hours.


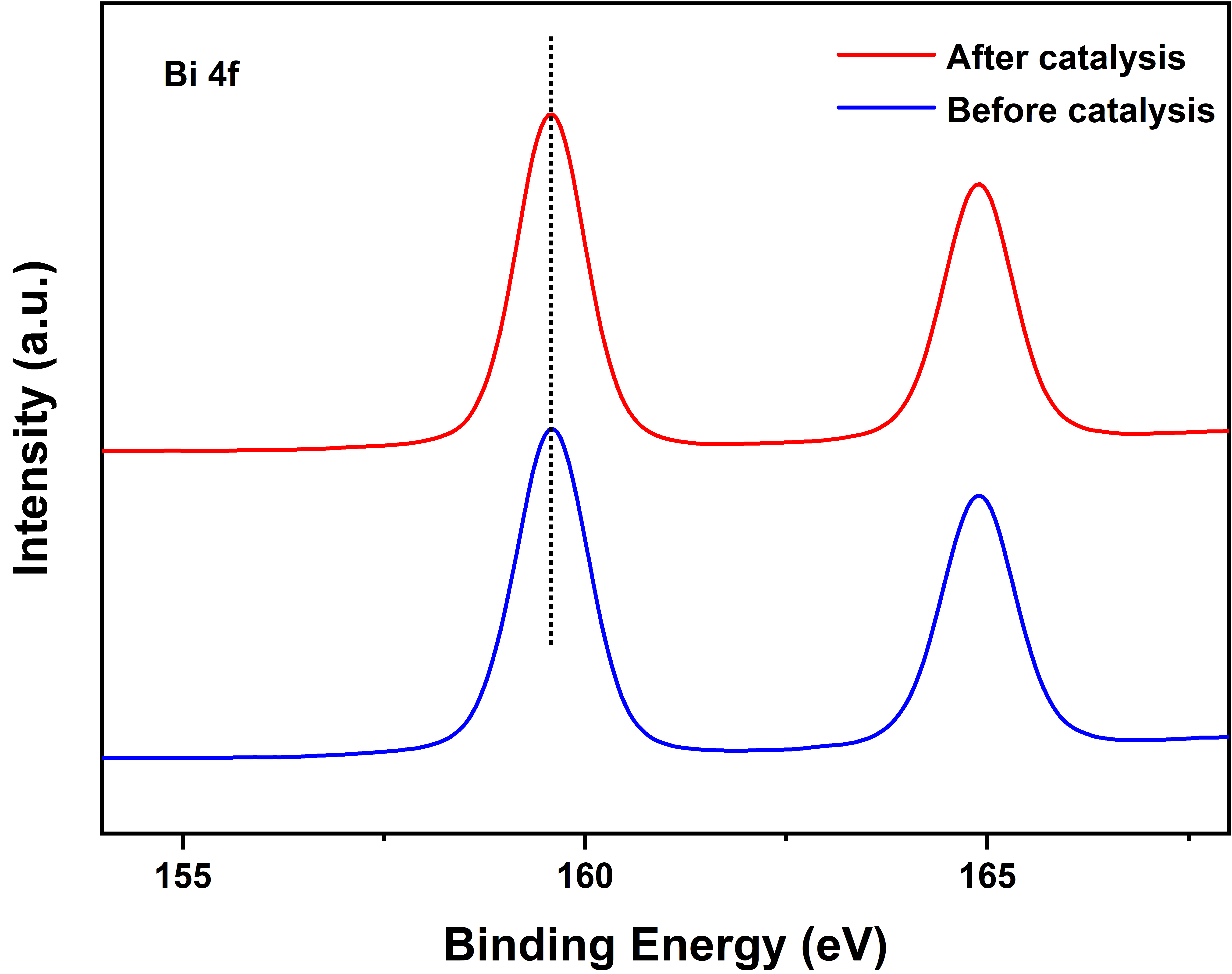


**Figure S13.** The XPS spectra of Bi 4f for BiOBr-(010) before and after catalysis.


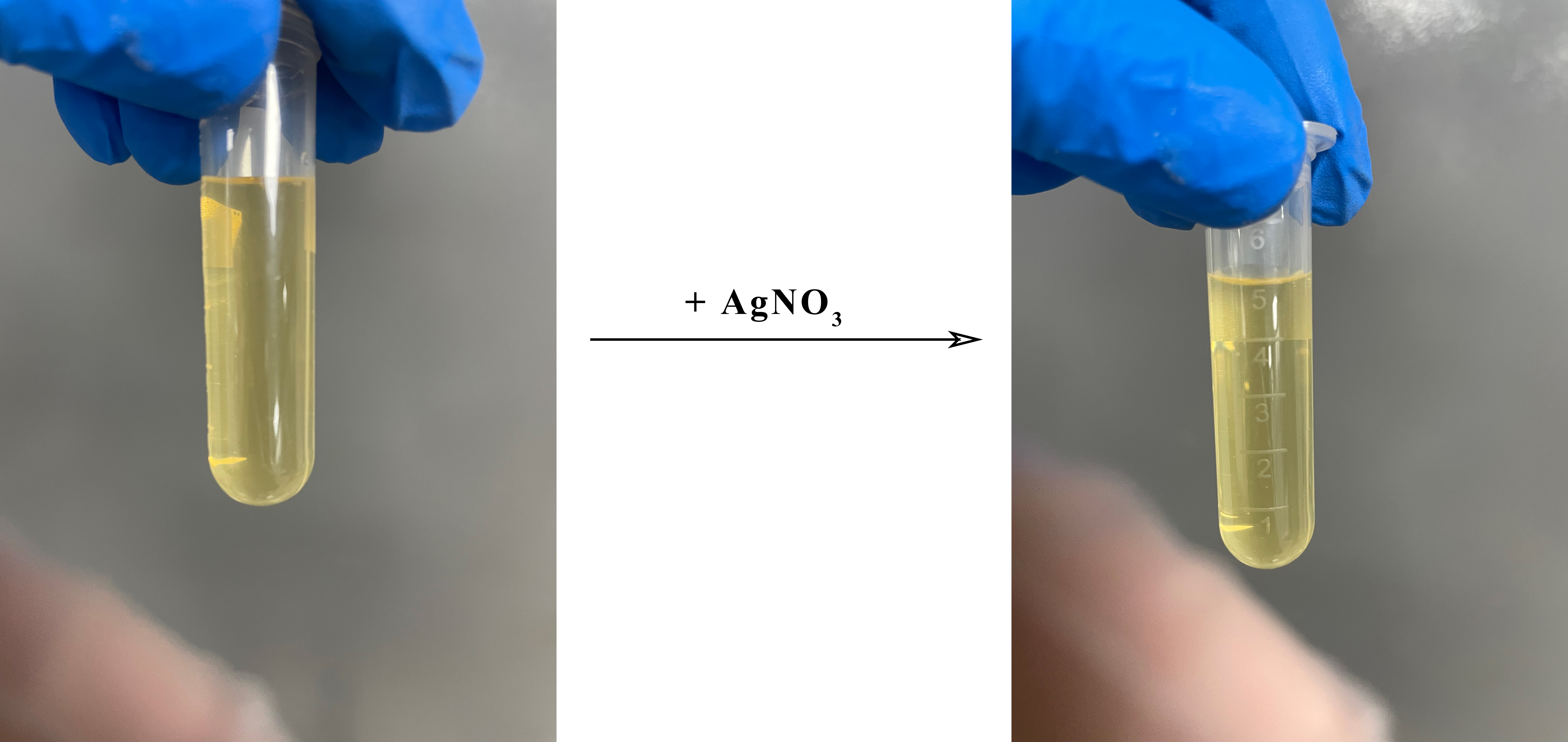


**Figure S14.** Detection experiments for leaching of Br ions. For the leaching of Br ions, we added a certain amount of AgNO_3_ to the supernatant after the reaction, as shown in Figure S14, no precipitation was produced, indicating that there was no leaching of Br ions after the reaction. In addition, no Br ions were detected in the supernatant by ICP-AES.


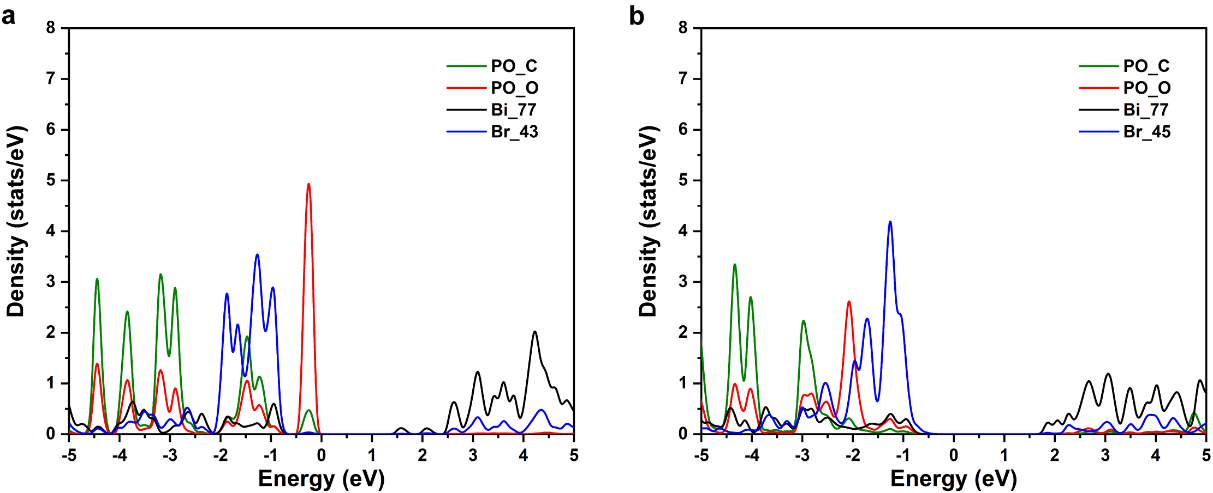


**Figure S15.** Calculated density of states of PO adsorbed on (a). (001)-, and (b). (010)- facet of BiOBr. 77 and 43 are the atom numbers in the BiOBr model in the DFT calculation, Bi_77 and Br_43 are the closest Bi atom and the closest O atom to the PO molecule in the BiOBr slab, respectively.


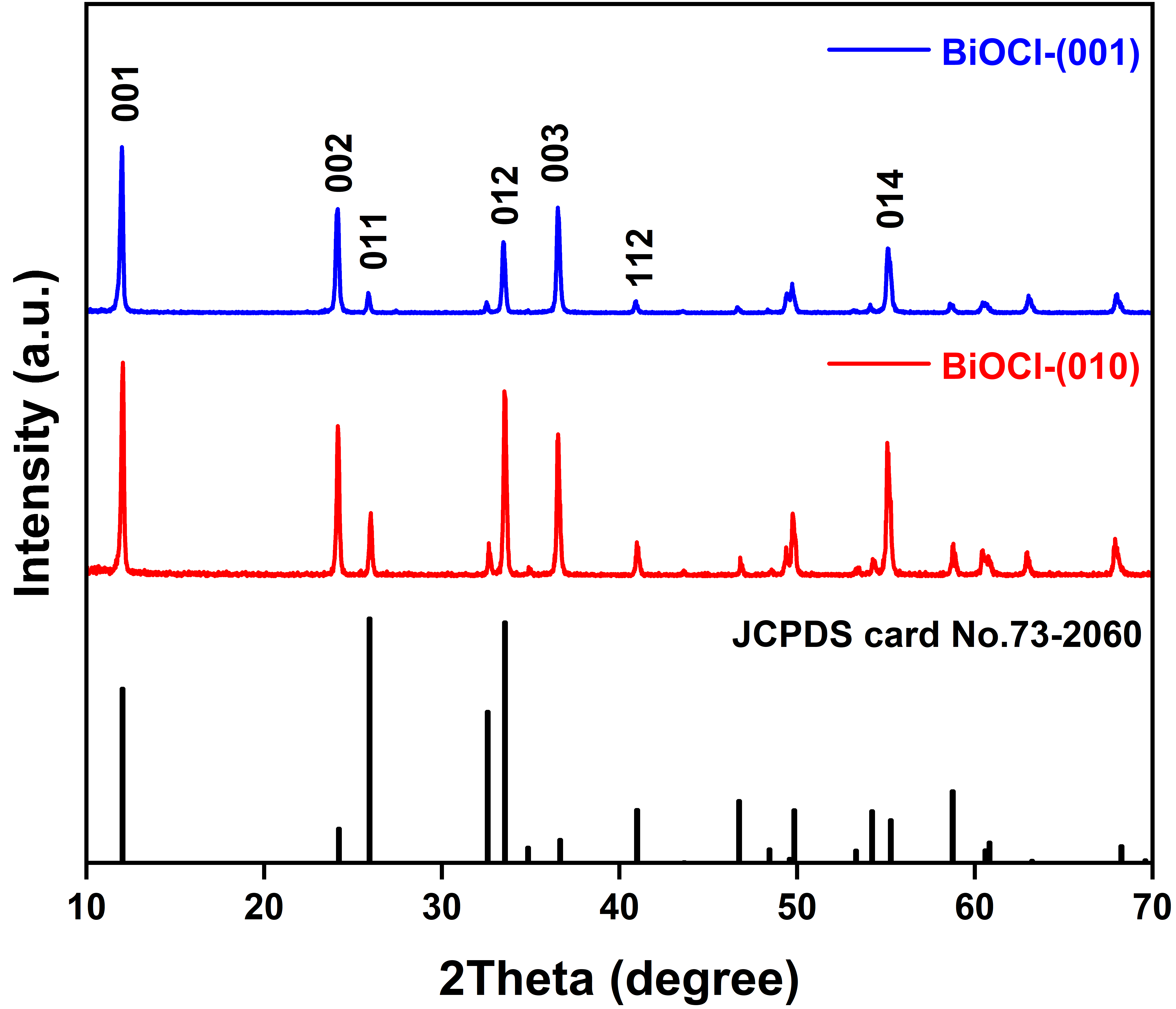


**Figure S16.** XRD patterns of the BiOCl samples.


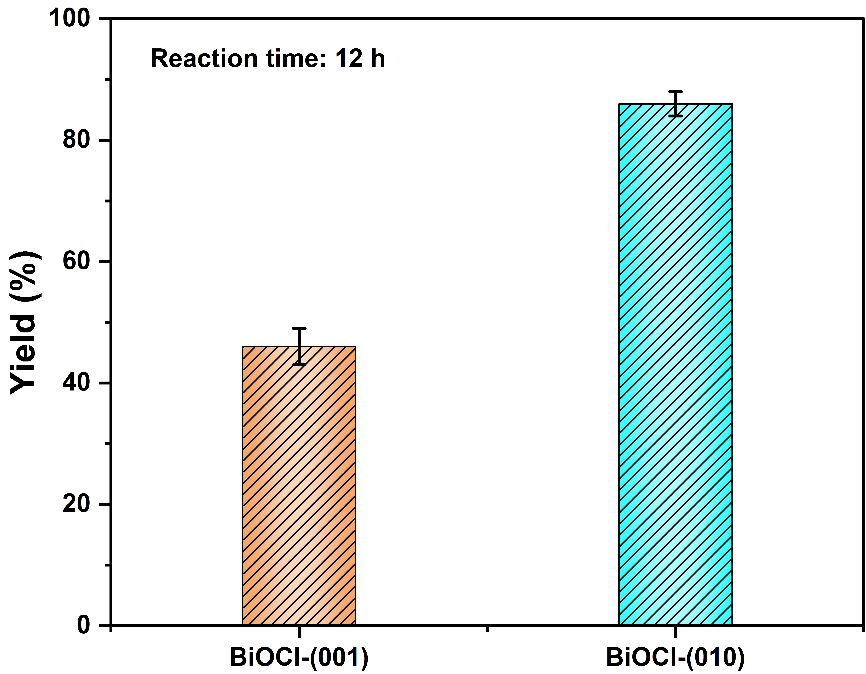


**Figure S17.** Performances of BiOCl samples for CO_2_ cycloaddition with PO. Reaction condition: CO_2_ pressure of 8 bar at 423 K.


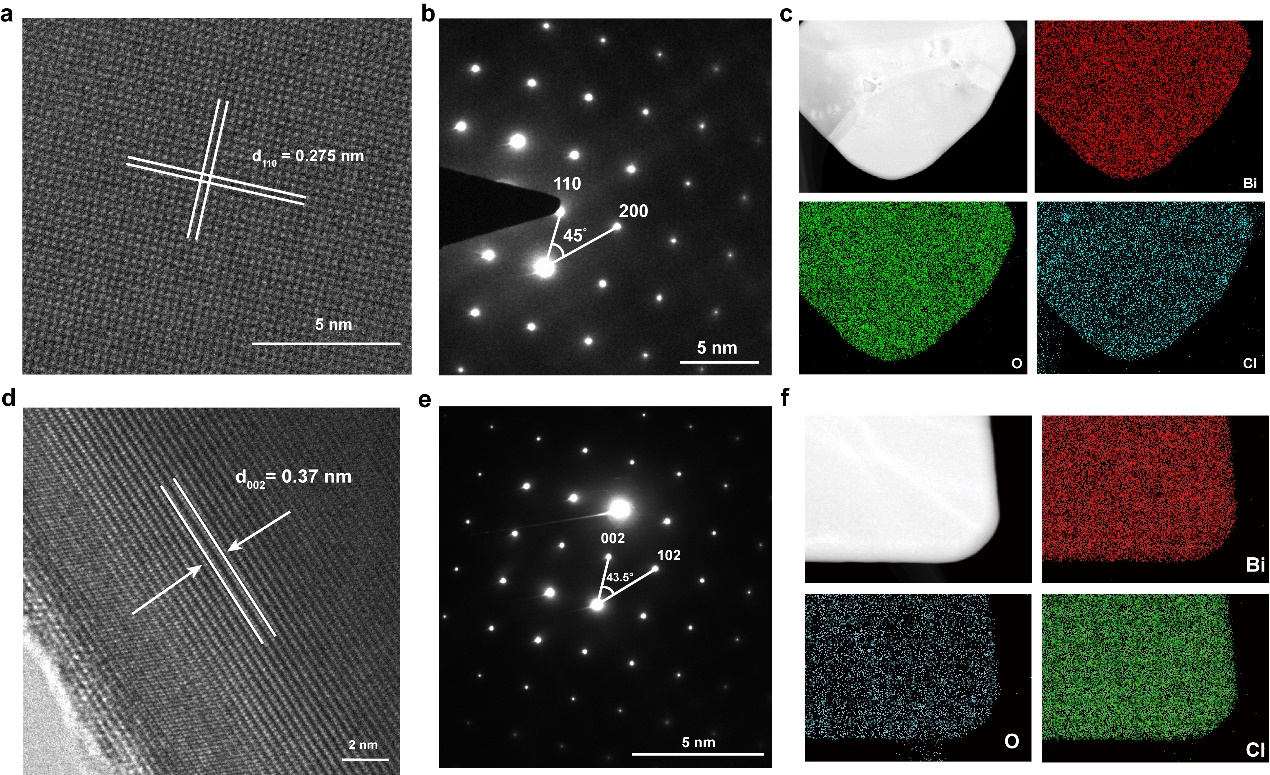


**Figure S18.** HRTEM, SAED and corresponding EDS mapping images of BiOCl-(001) (a-c) and BiOCl-(010) (d-f).

**Table S1.** **Cycloaddition of Epoxides with CO_2_^a^.**

| Entry | Substrate | Cat. | Con.(%) | Sel.(%) | Yie.(%) |
| --- | --- | --- | --- | --- | --- |
| 1 |  | - | 10 | 99 | 10 |
| 2 |  | BiOBr-(010) | 85 | 99 | 85 |
| 3 |  | BiOBr-(001) | 45 | 99 | 45 |
| 4 | 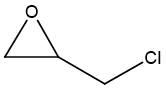 | BiOBr-(010) | 91 | 98 | 89 |
| 5 |  | BiOBr-(010) | 90 | 99 | 90 |
| 6 | 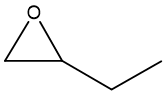 | BiOBr-(010) | 72 | 99 | 72 |
| 7 |  | BiOBr-(010) | 74 | 95 | 73 |
| 8 |  | BiOBr-(010) | 10 | 99 | 10 |

^a^Reaction conditions: epoxide (4.5 mL), catalyst (50 mg), DMF (13.5 mL), CO_2_ pressure (8 bar), 423 K, 12 h.

**Table S2.** Absorption energy of CO_2_, PO and PC on (001) and (010) facets of BiOBr.

| Adsorption  energy (eV) | CO_2_ | PO | PC |
| --- | --- | --- | --- |
| BiOBr-(001) | **-0.1532** | **-0.3974** | **-0.3989** |
| BiOBr-(010) | **-0.2281** | **-0.6755** | **-0.8298** |

**Table S3.** Bader charge analysis of CO_2_, PO and PC on (001) and (010) facets of BiOBr.

| Electron get | CO_2_ | PO | PC |
| --- | --- | --- | --- |
| BiOBr-(001) | **0.0194** | **0.0082** | **0.0324** |
| BiOBr-(010) | **0.0173** | **-0.0407** | **-0.0012** |

**Table S4.** **Cycloaddition of CO_2_ and PO using different catalysts under various reaction conditions without addition of co-catalyst.**

| Catalyst | $P_{CO_{2}}$(bar) | Temp. (°C) | Time (h) | PC Yield (%) | TON | Ref. |
| --- | --- | --- | --- | --- | --- | --- |
| BiOBr-(010) | 8 | 150 | 12 | 85 | 336 | - |
| ZIF-78 | 10 | 150 | 15 | 54 | 76 | (*3*) |
| Zn-C_3_N_4_ | 20 | 130 | 5 | 7 | 9 | (*4*) |
| Al-CPOP | 1 | 120 | 24 | 67 | 67 | (*5*) |
| GOPCoCl | 18 | 120 | 12 | 7.6 | 298 | (*6*) |
| UMCM-1-NH2 | 12 | 120 | 24 | 91 | 142 | (*7*) |
| mp-C_3_N_4_ | 25 | 140 | 6 | 12.4 | 8 | (*8*) |
| Zn-TiO_2_ | 20 | 120 | 8 | 13.7 | 5 | (*9*) |

**Table S5.** Comparison of performances of traditional metal oxides for cycloaddition of CO_2_ and PO.

| Catalyst | $P_{CO_{2}}$(bar) | Temp. (°C) | Time (h) | PC Yield (%) | Ref. |
| --- | --- | --- | --- | --- | --- |
| BiOBr-(010) | 8 | 150 | 12 | 85 | - |
| MgO | 80 | 150 | 15 | 32.1 | (*10*) |
| CaO | 80 | 150 | 15 | 0.8 | (*10*) |
| ZnO | 80 | 150 | 15 | 8.7 | (*10*) |
| ZrO_2_ | 80 | 150 | 15 | 10.9 | (*10*) |
| La_2_O_3_ | 80 | 150 | 15 | 54.1 | (*10*) |
| Al_2_O_3_ | 80 | 150 | 15 | 6.6 | (*10*) |
| Nb_2_O_5_ | 50 | 150 | 12 | 88 | (*11*) |

**References**

1. H. Wang, S. Chen, D. Yong, X. Zhang, S. Li, W. Shao, X. Sun, B. Pan, Y. Xie, Giant Electron–Hole Interactions in Confined Layered Structures for Molecular Oxygen Activation. *J. Am. Chem. Soc.* **139**, 4737–4742 (2017).

2. M. Shi, G. Li, J. Li, X. Jin, X. Tao, B. Zeng, E. A. Pidko, R. Li, C. Li, Intrinsic Facet‐Dependent Reactivity of Well‐Defined BiOBr Nanosheets on Photocatalytic Water Splitting. *Angew. Chem. Int. Ed.* **59**, 6590–6595 (2020).

3. Y.-F. Lin, K.-W. Huang, B.-T. Ko, K.-Y. A. Lin, Bifunctional ZIF-78 heterogeneous catalyst with dual Lewis acidic and basic sites for carbon dioxide fixation via cyclic carbonate synthesis. *J. CO_2_ Util.* **22**, 178–183 (2017).

4. X. Wang, M. S. Liu, L. Yang, J. W. Lan, Y. L. Chen, J. M. Sun, Synthesis of Zn Modified Carbon Nitrides Heterogeneous Catalyst for the Cycloaddition of CO_2_ to Epoxides. *ChemistrySelect*. **3**, 4101–4109 (2018).

5. Y.-F. Lin, K.-W. Huang, B.-T. Ko, K.-Y. A. Lin, T.-T. Liu, J. Liang, Y.-B. Huang, R. Cao, A bifunctional cationic porous organic polymer based on a Salen-(Al) metalloligand for the cycloaddition of carbon dioxide to produce cyclic carbonates. *Chem. Commun.* **52**, 13288–13291 (2017).

6. J. Liu, A. Wang, H. Jing, W. Wang, Y. Wang, C. Li, L. Yan, M. Jiang, Y. Ding, R. B. Mujmule, W.-J. Chung, H. Kim, F. Gou, J. Liu, N. Ye, X. Jiang, C. Qi, Cobalt-porphyrin modified graphene oxide as a heterogeneous catalyst for solvent-free CO_2_ fixation to cyclic carbonates. *J. CO_2_ Util.* **48**, 101534 (2021).

7. Y.-F. Lin, K.-W. Huang, B.-T. Ko, K.-Y. A. Lin, T.-T. Liu, J. Liang, Y.-B. Huang, R. Cao, R. Babu, A. C. Kathalikkattil, R. Roshan, J. Tharun, D.-W. Kim, D.-W. Park, Dual-porous metal organic framework for room temperature CO_2_ fixation via cyclic carbonate synthesis. *Green Chem.* **18**, 232–242 (2017).

8. J. Liu, A. Wang, H. Jing, W. Wang, Y. Wang, C. Li, L. Yan, M. Jiang, Y. Ding, R. B. Mujmule, W.-J. Chung, H. Kim, F. Gou, J. Liu, N. Ye, X. Jiang, C. Qi, J. Xu, F. Wu, Q. Jiang, Y.-X. Li, Mesoporous carbon nitride grafted with n-bromobutane: a high-performance heterogeneous catalyst for the solvent-free cycloaddition of CO_2_ to propylene carbonate. *Catal. Sci. Amp Technol.* **5**, 447–454 (2021).

9. J. Liu, A. Wang, H. Jing, TiO2-based green heterogeneous catalysts for the cycloaddition of CO_2_ to epoxides. *Chin. J. Catal.* **35**, 1669–1675 (2014).

10. B. M. Bhanage, S. Fujita, Y. Ikushima, M. Arai, Synthesis of dimethyl carbonate and glycols from carbon dioxide, epoxides, and methanol using heterogeneous basic metal oxide catalysts with high activity and selectivity. *Appl. Catal. Gen.* **219**, 259–266 (2001).

11. M. Aresta, Nb(V) compounds as epoxides carboxylation catalysts: the role of the solvent. *J. Mol. Catal. Chem.* **204–205**, 245–252 (2003).
